# Effect of Hexavalent Chromium [Cr(VI)] on Phytoremediation Potential and Biochemical Response of Hybrid Napier Grass with and without EDTA Application

**DOI:** 10.3390/plants8110515

**Published:** 2019-11-17

**Authors:** Bhagat Kanwar Ram, Ying Han, Gang Yang, Qin Ling, Faqin Dong

**Affiliations:** 1 School of Life Sciences and Engineering, Southwest University of Science and Technology, Mianyang 621010, China; msdo_sindh@yahoo.com (B.K.R.); hanying@swust.edu.cn (Y.H.); yanggang903@swust.edu.cn (G.Y.); 2 Department of Energy and Environment, Faculty of Agricultural Engineering, Sindh Agriculture University, Tandojam 70060, Pakistan; 3 School of Environment and Resources, Southwest University of Science and Technology, Mianyang 621010, China; lingqinchn@163.com

**Keywords:** hexavalent Cr, phytoremediation, hybrid napier, *Pennisetum*, EDTA

## Abstract

Hexavalent chromium [Cr(VI)] contamination has become an emergent concern in China. Previous field investigations have found that hybrid Napier grass is widely distributed in Cr(VI) contaminated areas. This study investigated the phytoremediation potential and biochemical response of hybrid Napier grass (*Pennisetum americanus* L. × *Pennisetum purpureum* Schumach) grown in soil contaminated with Cr(VI) (0, 20, 40, and 60 mg kg^−1^) with and without Ethylene diamine tetra acetic acid (EDTA) (4 mM) application. The results indicated that root length, shoot height, dry weight, leaf area, chlorophyll, and photosystem II (PSII) parameters viz.; apparent electron transport rate (ETR), effective quantum yield of PSII (ΦPSⅡ), maximal PSII photochemical efficiency (Fv/Fm), potential activity of PSII (Fv/Fo), photochemical quenching (qP), and non-photochemical quenching (qN) decreased with the increasing Cr(VI) concentration. EDTA application further aggravated reduction of dry biomass and photosystem II. The concentration and the accumulation of Cr in shoot and root, and both the bioaccumulation factor (BAF) and transfer factor (TF) increased with increasing Cr(VI) concentrations and further enhanced with EDTA application. Though the Cr(VI) and Ethylene diamine tetra acetic acid (EDTA) stress reduced tolerance, but, even at highest Cr(VI) concentration, plant could exhibited strong resistance, as evidenced by increase in superoxide dismutase (SOD), peroxidase (POD), and catalase (CAT) activities. Hybrid Napier grass, due to its BAF > 1 and a TF < 1, would be applicable for Cr phytostabilization. Moreover, limiting metal transport to aerial parts of plant would prevent animal’s ingestion, restrict soil mobility, and consequently reduce transmission across the food chain.

## 1. Introduction

Leather, printing and dyeing industries are very well developed in different parts of the world, including China, which mainly exist in the form of small and medium-sized enterprises. Large concentrations of hexavalent chromium [Cr(VI)] are discharged into the environment, which poses a great threat to the surrounding ecological environment due to the imperfection of production process and wastewater treatment process [1]. However, the natural, average, and background levels of Cr in soils vary greatly in different regions of the world, as shown in Table 1 [2]. The Cr(VI) occurs in strong oxidizing forms and it is very toxic to plant, animal, and human, because of its high solubility and mobility [3]. Ethylene diamine tetra acetic acid (EDTA) is one of the common chelating agents, which enhances metal uptake, increases its transport from roots to shoot, and alters the metal speciation and phytotoxicity [4,5,6]. EDTA can disturb the concentration balance of Cr(VI) in the liquid and solid phases of soil to form mobile compounds, which can be leached with water or absorbed by plants by chelating or coordinating some difficult-to-move Cr [7]. Some resistant plants can still grow and reproduce there despite the harsh environment around these Cr(VI) contaminated factories.

Our previous field investigations have found that hybrid Napier grass (*Pennisetum americanum* L. × *Pennisetum purpureum* Schumach) widely distributed in the areas contaminated by different levels of Cr(VI), which indicated that the plant had strong resistance to Cr pollution, but its ability to absorb Cr was not clear. Besides, the information regarding the effects of different concentrations of Cr(VI) with and without EDTA application on the phytoremediation potential and physiological characteristics of hybrid Napier grass is lacking. Such information would be critical in the development of hybrid Napier grass as a phytoremediation plant in Cr-contaminated areas. Previous studies indicated that Cr(VI) stress has a stimulating effect on the growth of most plant seedlings at low concentrations, whereas it has an inhibiting effect on plant at high concentrations [18,19,20,21]. A low concentration of Cr(VI) can increase the net photosynthetic rate and promote the growth of plants by enhancing the electron transfer activity of PSII. It can increase the proportion of pith and epidermis in roots and promote the growth of roots and root hairs [18]. Whereas, a high concentration of Cr(VI) hinders water transport, reduces transpiration, affects root uptake of mineral elements, and interferes with enzymatic reactions in plants, which results in plant dwarfing, leaf yellowing and shedding, and biomass reduction [19,20,21,22,23]. In addition, Bareen and Tahira [24] found that seven different cultivated plant species absorbed more Cr under EDTA application. Although the phytotoxicity and remediation potential under soil Cr(VI) stress has been investigated in many plant species, the *Pennisetum* species have been given less attention. Thus, it is crucial to understand the response of the native plants distributed at Cr contaminated area to develop potential Cr hyperaccumulator or Cr-tolerant plants for remediating Cr-contaminated soils or the rehabilitation of vegetation.

*Pennisetum* species belong to family Poaceae, which grow annually or perennially. There are about 140 species in the world, most of which are native to Africa. In China, they are mainly distributed in Northeast, North, East, Central South, and Southwest of China. Hybrid Napier grass is a triploid hybrid that is produced by crossing diploid *Pennisetum americanus* L. with tetraploid elephant grass (*Pennisetum purpureum* Schumach). Hybrid Napier grass species are more vigorous and resistant to unfavorable environment [25,26]; however, its mechanism of tolerance has not been fully understood. In addition, its tolerance and accumulation characteristics of heavy metals in polluted soils remain to be discovered. Therefore, the objectives of the present study were to evaluate the effect of Cr(VI) with and without EDTA application on the phytoremediation potential, growth performance, photosynthetic efficiency, and antioxidant enzyme activity in native hybrid Napier grass to develop potential Cr hyperaccumulator or Cr-tolerant plants for remediating Cr-contaminated soils or rehabilitation of vegetation.

## 2. Results

### 2.1. Growth Characteristics of Hybrid Napier Grass

**Figure 1:**
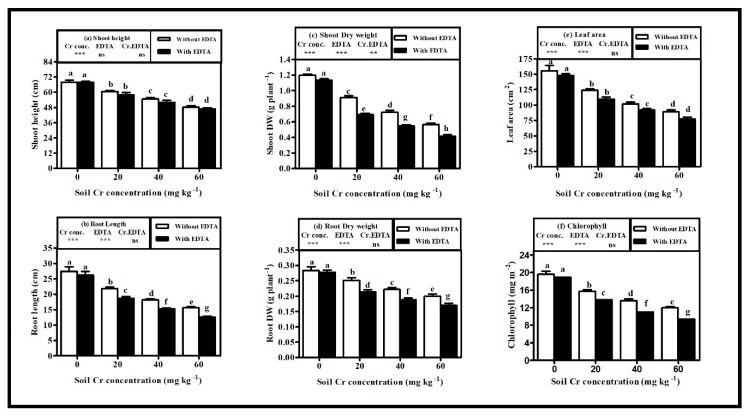
Influence of various Cr(VI) concentrations in soil on (**a**) shoot height, (**b**) root length, (**c**) shoot dry weight, (**d**) root dry weight, (**e**) leaf area, and (**f**) chlorophyll content of hybrid Napier grass with and without ethylene diamine tetra acetic acid (EDTA) application. Bars with dissimilar letters are significantly different at *p* < 0.05 from Tukey’s test. Values are means ± SE, *n* = 5. ** *p* < 0.001; *** *p* ≤ 0.001. ns: non-significant, Cr conc.: soil Cr(VI) concentration effect; EDTA: EDTA effect and Cr conc. EDTA: Cr(VI) conc. × EDTA interaction effect.

The Cr(VI) treatment produced dose-dependent effects on plant growth in both non-EDTA treated plants (NET-plants) and EDTA treated plants (ET-plants) and the maximum effects were noticed at highest dose of chromium Cr60 (60 mg kg^−1^) in soil as compared with their corresponding controls (Cr0 i.e., 0 mg kg^−1^ of Cr(VI) in soil) (Figure 1a–f). Shoot height (SH), root length (RL), and leaf area (LA) were decreased (*p* < 0.005) by 28.89%, 31.95%, and 42.82% in NET-plants and by 30.74%, 41.79%, and 47.78% in ET-plants, respectively, at Cr60 when compared with their corresponding controls (Figure 1a,b,e). Dry weights (DW) of shoot and root decreased (*p* < 0.005) by 53.33% and 29.58%, respectively, in NET-plants and by 26.79% and 38.85%, respectively, in ET-plants, at Cr60 when compared with their corresponding controls (Figure 1c,d).

Comparison between two treatments showed that the SH, RL, shoot DW, root DW, and LA decreased by 2.93%, 19.82%, 26.79%, 15%, and 12.94% in ET-plants as compared with NET-plants at Cr60 (Figure 1a–e). However, the above measured parameters were statistically non-significant (*p* > 0.05) between the two groups, except for DW of root and shoot, which showed a significant decrease (*p* < 0.05) in ET-plants as compared with NET-plants.

### 2.2. Cr Accumulation and Phytoremediation Potential

The Cr concentration and accumulation in root and shoot of hybrid Napier grass increased with increasing Cr(VI) concentrations in soil (Table 2). Generally, the Cr concentration and accumulation was higher in the root than in shoot. The Cr concentration and accumulation increased by 34.73% and 6.94% in roots and by 230.29% and 103.81% in shoot of NET-plants, respectively, at Cr60 as compared with Cr20 (20 mg kg^−1^ of Cr(VI) in soil). The Cr concentration and accumulation increased by 99.86% and 58.89% in roots and by 234.7% and 101.64% in shoot of ET-plants, respectively, at Cr60 when compared with Cr20. EDTA application significantly increased Cr concentration and accumulation in shoot, whereas decreased their concentrations in roots (Table 2). The maximum increase in Cr concentration and accumulation occurred by 114.32% and 59.28%, respectively, in shoot of ET-plants as compared with NET-plants at Cr60. Whereas the maximum decrease in Cr concentration and accumulation in roots was observed by 81.52% and 114.3%, respectively, at Cr40 in ET-plants as compared with NET-plants.

The bioaccumulation factor (BAF) and the transfer factor (TF) were measured to estimate the transfer of Cr from treated soil to the plant to assess the phytoremediation potential of plant (Table 2). The BAF values were > 1 and further increased with increasing Cr(VI) concentrations in soil. The maximum BAF values were 1.61 and 2.86 in NET-plants and ET-plants at Cr40 (40 mg kg^−1^ of Cr(VI) in soil), respectively. The EDTA application increased the BAF by 114.75% at Cr60 in ET-plants as compared with NET-plants. Though the TF increased with increasing Cr(VI) concentration in soil, the overall values for TF were < 1. The maximum values for TF were 0.15 at Cr60 in NET-plants and 0.44 at Cr40 in ET-plants, respectively. Moreover, the EDTA application increased Cr transport from root to shoot and the maximum increase in TF was observed by 233.33% at Cr20 in ET-plants as compared with NET-plants.

### 2.3. Tolerance Index

The Cr(VI) stress reduced the tolerance indices (TI) of hybrid Napier grass with increasing Cr(VI) concentrations in soil (Table 3). A maximum decrease in TI of root and shoot was observed by 29% and 53% in NET-plants and by 39% and 63% in ET-plants at Cr60, respectively, when compared with their corresponding controls (TI = 1). The EDTA application caused a further decrease in TI and the reduction was 27% and 16.4% higher in the shoot and root of ET-plants as compared with NET-plants. Moreover, the Cr(VI) toxicity was higher in the shoot than in root of hybrid Napier grass.

### 2.4. Chlorophyll Content and Photosynthetic Efficiency

The Cr(VI) contamination in soil caused a concentration dependent reduction in the leaf chlorophyll content of hybrid Napier grass (Figure 1f) and the maximum decrease (*p* < 0.001) was observed by 38.98% in NET-plants and by 50.68% in ET-plants at Cr60 when compared with their corresponding controls. However, the reduction of chlorophyll content was further escalated with EDTA application in Cr(VI) contaminated soil. The EDTA application led to a reduction in chlorophyll content by 21.74% at Cr60 in ET-plants when compared with NET-plants.

**Figure 2:**
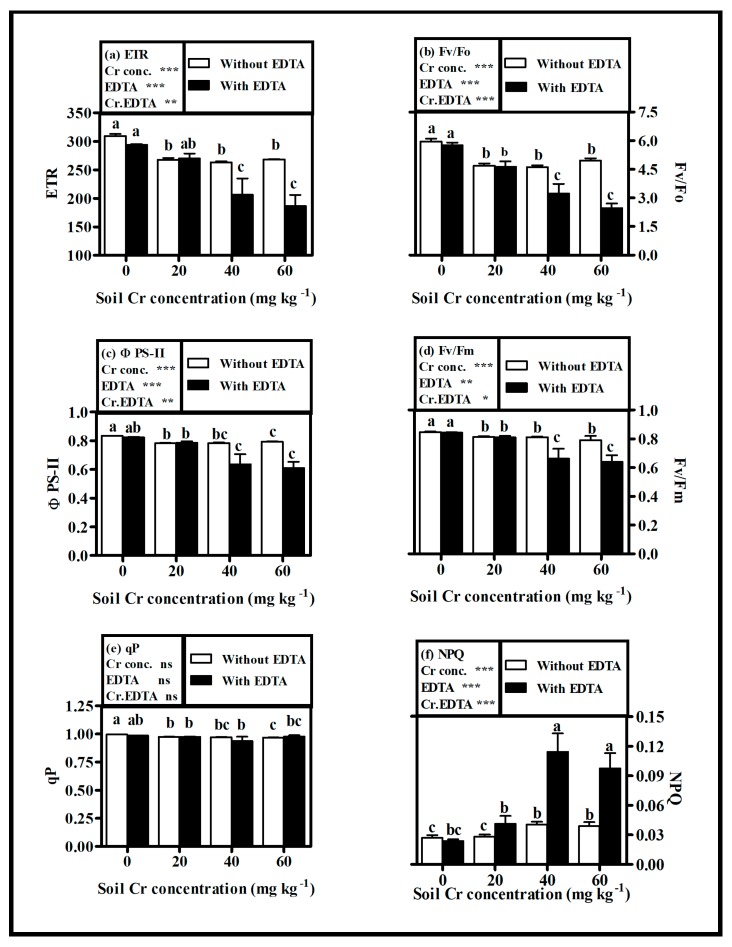
Influence of various Cr(VI) concentrations in soil on chlorophyll-α fluorescence parameters: (**a**) apparent electron transport rate (ETR), (**b**) potential activity of PSII (Fv/Fo), (**c**) effective quantum yield of PSII (ΦPSII) (**d**) maximal PSII photochemical efficiency (Fv/Fm) (**e**) photochemical quenching (qP) and (**f**) non-photochemical quenching (NPQ or qN) of hybrid Napier grass with and without EDTA application. Bars with dissimilar letters are significantly different at *p* < 0.05 from Tukey’s test. Values are means ± SE, *n* = 5. * *p* < 0.05; ** *p* < 0.001; *** *p* ≤ 0.001. ns: non-significant, Cr conc.: soil Cr(VI) concentration effect; EDTA: EDTA effect and Cr conc. EDTA: Cr(VI) conc. × EDTA interaction effect.

Photosynthetic capacity of plant, such as maximum quantum yield (Fv/Fm), efficiency of water-splitting complex (Fv/Fo), effective photochemical quantum yield of PSII (ΦPSII), electron transport (ETR), photochemical quenching (qP), and non-photochemical fluorescence quenching (qN), was measured (Figure 2a–f). In NET-plants, Cr(VI) stress caused slight reduction (*p* < 0.05) by 7%, 5.2% and 2.8% in Fv/Fm, ΦPSII and qP at Cr60, respectively, as compared with control. Whereas, ETR, Fv/Fo, and qN were moderately affected by Cr(VI) stress. ETR and Fv/Fo were decreased (*p* < 0.05) by 15.6% and 20% and qN increased by 43.9%, respectively, at Cr60 when compared with control. Moreover, no significant difference (*p* > 0.05) was observed among various Cr(VI) doses on the photosynthetic efficiency of NET-plants.

On the other hand, Cr(VI) produced dose-dependent effects on PSII efficiency in ET-plants, except for qP, which showed no significant difference (*p* > 0.05) among the treatments. The maximum decrease (*p* < 0.05) was observed by 31.6%, 134%, 35%, and 57.5% in Fv/Fm, Fv/Fo, ΦPSII, and ETR at Cr60, respectively, as compared with control. Nevertheless, qN increased by 314% at Cr60 when compared with control. The addition of EDTA along with Cr(VI) in soil fortified the toxic effects on photosynthetic efficiency in plant. The EDTA application caused a further decrease in Fv/Fm, Fv/Fo, ΦPSII, and ETR by 19.1%, 50.4%, 23.1%, and 30.3%, and increase in qN by 59.8% at Cr60, respectively, in ET-plants as compared with NET-plants.

### 2.5. Nitrogen (N) and Sulfur (S) Status in Hybrid Napier Grass

**Figure 3:**
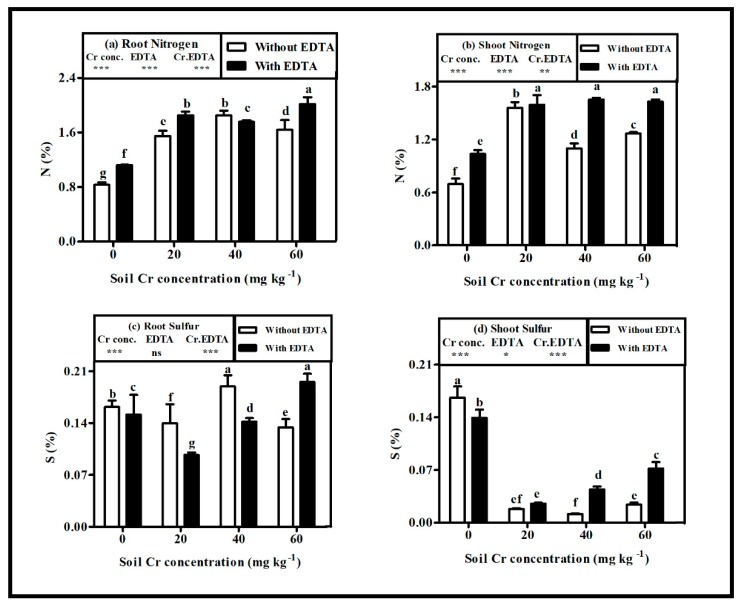
Influence of various Cr(VI) concentrations in soil on element levels (%) of (**a**) root nitrogen, (**b**) shoot nitrogen, (**c**) root sulfur, and (**d**) shoot sulfur of hybrid Napier grass with and without EDTA application. Bars with dissimilar letters are significantly different at *p* < 0.05 from Tukey’s test. Values are means ± SE, *n* = 5. * *p* < 0.05; ** *p* < 0.001; *** *p* ≤ 0.001. ns: non-significant, Cr conc.: soil Cr(VI) concentration effect; EDTA: EDTA effect and Cr conc. EDTA: Cr(VI) conc. × EDTA interaction effect.

Irrespective of dosage, Cr(VI) stress caused significant (*p* < 0.05) alterations in contents (%) of nitrogen (N) and sulfur (S) in root and shoot of plant (Figure 3a–d). The N content increased by 55.45% and 54.2% in root and shoot of NET-plants at Cr40 and Cr20, respectively, whereas the S content decreased by 26.71% and 93.6% in root and shoot of NET-plants at Cr20 and Cr40, respectively, when compared to control. In ET-plants, maximum increase in N content was observed by 47.7% in root at Cr60 and by 39.75% in shoot at Cr 20, respectively, as compared with the control. The S content in root first decreased by 17.6% at Cr20 and then maximum increased by 81.8% at Cr60 when compared with the control. Nevertheless, the S content in shoot decreased at all Cr(VI) concentrations and the maximum decrease was observed by 82.6% at Cr20 as compared with control.

EDTA application showed a variable response to N and S contents in the root. The N content in root increased by approximately 18% at Cr20 and Cr60, respectively, and decreased by 9.4% at Cr40 in ET-plants when compared with NET-plants. The S content in root first decreased by approximately 25% at Cr20 and Cr40, respectively, and then increased by 28.63% at Cr60, their corresponding controls. Whereas, the EDTA application increased N and S contents in shoot at all Cr(VI) concentrations and the maximum increase in N and S was observed by 50.74% and 284.35% at Cr40, respectively, in ET-plants when compared with NET-plants.

### 2.6. Oxidative Stress

**Figure 4:**
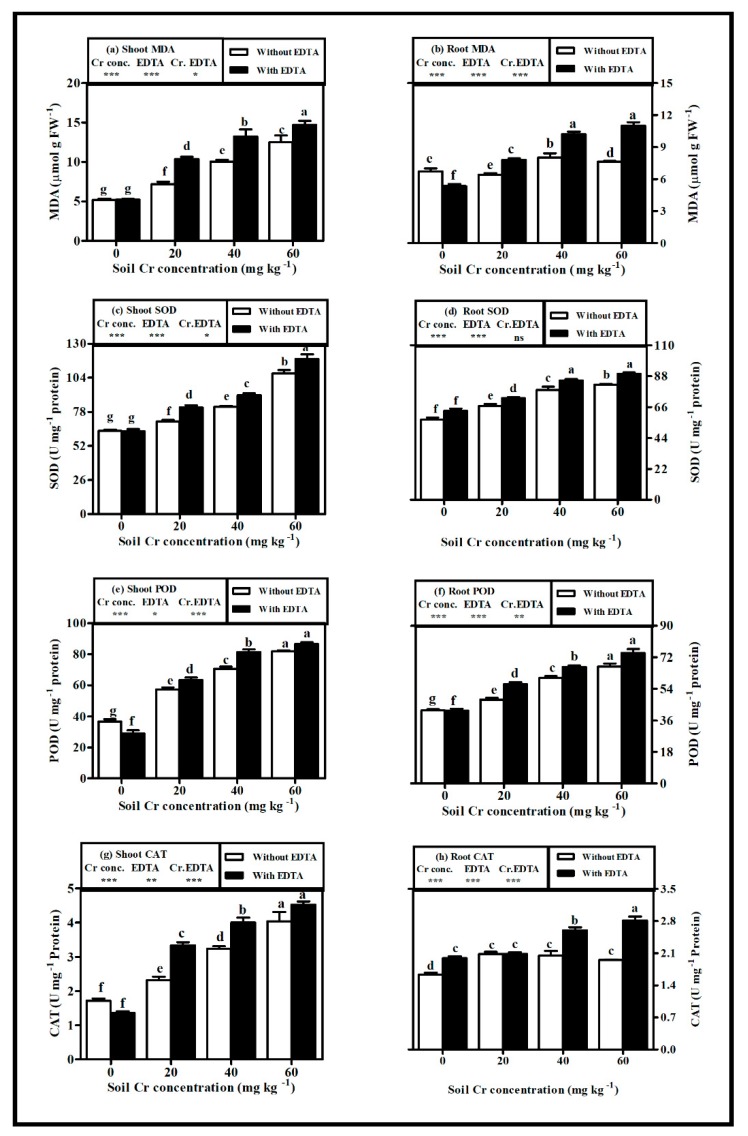
Influence of various Cr(VI) concentrations in soil on (**a**) malondialdehyde (MDA) level in shoot, (**b**) MDA level in root, (**c**) superoxide dismutase (SOD) activity in shoot, (**d**) SOD activity in root (**e**) peroxidase (POD) activity in shoot, (**f**) POD activity in root (**g**) catalase (CAT) activity in shoot and (**h**) CAT activity in root of hybrid Napier grass with and without EDTA application. Bars with dissimilar letters are significantly different at *p* < 0.05 from Tukey’s test. Values are means ± SE, *n* = 5. * *p* < 0.05; ** *p* < 0.001; *** *p* ≤ 0.001. ns: non-significant, Cr conc.: soil Cr(VI) concentration effect; EDTA: EDTA effect and Cr conc. EDTA: Cr(VI) conc. × EDTA interaction effect.

Decreased tolerance was associated with increased oxidative stress in hybrid Napier grass that was grown in soil with varying concentrations of Cr(VI) stress. (Figure 4a–h). Overall, the malondialdehyde (MDA) level (Figure 4a,b) and activities of anti-oxidative enzymes, including superoxide dismutae (SOD), peroxidase (POD), and catalase (CAT) (Figure 4c–h), invariably increased with all tested Cr(VI) doses. The respective maximum increase in oxidative stress markers in the root and shoot of NET-plants were observed as MDA (19.21% and 140.5%), SOD (43.29% and 123.37%), POD (59.36% and 69.1%), and CAT (27.17% and 135.82%) at Cr60 as compared with control. Whereas, in ET-plants, maximum increase in root and shoot (MDA 106.64% and 180.69%), SOD (41.52% and 198.2%), POD (78.41% and 86.93%), and CAT (41.45% and 232.99%) were observed at Cr60 when compared with control.

EDTA application aggravated the effects of Cr(VI) on MDA level and antioxidant enzyme activities in the root and shoot of the plant. The MDA level, SOD, and CAT activities increased by 44.29%, 9.5%, and 44.3%, respectively, at Cr60, and the POD activity increased by 18.3% at Cr20 in root of ET-plants as compared with that of NET-plants. Whereas, in shoot, the MDA level, SOD, and CAT activities increased by 43.9%, 15.2%, and 43.9%, respectively, at Cr20, and the POD activity increased by 15.2% at Cr40 in ET-plants when compared with that of NET-plants. Concurrent with increased toxicity, the oxidative stress was higher in the shoot than in root of both NET-plants and ET-plants.

## 3. Discussion

### 3.1. Plant Growth and Phytotoxicity

In our study, Cr(VI) negatively affected the growth of hybrid Napier grass and reduced the plant yield and biomass in dose-dependent manner. The adverse effects of Cr(VI) on plant growth have been well documented [27,28,29]. A reduction in biomass and plant yield is caused by the stunted growth of shoot and leaf, which might be due to the toxic effects of Cr(VI) on photosynthesis and it might be partially due to a reduced transport of water and nutrients from soil caused by reduced root growth in presence of Cr(VI) [22,28]. Reduced root growth might be due to tissue collapse resulting from the inhibition of proliferation and elongation of root consequently result in incapability of the roots to absorb water and nutrients from the medium [9,30].

As a consequence of biomass reduction, the Cr(VI) stress reduced the tolerance indices (TI) of hybrid Napier grass in dose-dependent manner and the application of EDTA enhanced the Cr(VI) toxicity in plant. The most significant effects of EDTA were observed on DW of root and shoot. Our findings are in accordance with Bareen et al. [31], who reported increased phytotoxic effects in sorghum (*Sorghum bicolor*) and pearl millet (*Pennisetum glaucum*) species that were treated with Cr(VI) and EDTA co-application than Cr(VI)-alone, and the most severe toxic effects were observed on root length. The inhibitory effects of EDTA on growth and the reduction of dry biomass have been reported in marigold (*Tagetes* sp.) [32]. The decrease in DW of root and shoot of the plant following EDTA application can be attributed to EDTA toxicity and Cr-EDTA chelant complex formation [31]. One of the reasons of negative effects of EDTA can be the impaired absorption of essential nutrients, such as Zn^2+^ and Ca^2+^, due to increased mobilization of heavy metal in the soil, which negatively impacts the cell wall elasticity and viscosity, reduce cell division and transpiration, and impair the cell membranes [31,32]. Moreover, the Cr(VI) toxicity was higher in the shoot than in root of hybrid Napier grass. Amin et al. [27] studied the effects of varying Cr(VI) contaminations (0.5–75 mg Cr kg^−1^ soil) in several plant species observed that toxic effects of Cr(VI) were greater in shoots than in roots of the plants.

### 3.2. Cr Accumulation and Phytoremediation Potential

In this study, Cr accumulation in the root and shoot increased with increasing Cr(VI) concentrations in soil and the maximum increase occurred at the highest concentration (Cr60). The amount of Cr(VI) uptake, transport, and accumulation in different organs of the plant vary with species and it depends upon the dosage and period of Cr(VI) treatment [22]. The seedlings of maize (*Zea mays*) cultivated in soil contaminated with 10 and 20 mg kg^−1^ Cr(VI) for 30 days accumulated 15.2 and 16.3 mg kg^−1^ of Cr, respectively [33]. Whereas, the exposure of gram (*Cicer arietinum* L.) seedlings to Cr(VI) stress at doses of 25, 50, and 75 ppm resulted in Cr accumulation of up to 0.2, 0.5, and 0.1 g kg^−1^ in roots, and 0.085, 0.2 and 0.05 g kg^−1^ in shoot, respectively [30]. Similarly, Cr accumulation ranged between 10–30 mg kg^−1^ DW in paddy (*Oryza sativa* L) seedlings that were treated with 2.5–200 mg L^−1^ Cr(VI) [34].

Cr accumulation was higher in roots than in the shoot of hybrid Napier grass. Huffman and Allaway [35] found that bean and wheat (*Triticum aestivum*) plants accumulated over 90% Cr in their roots, while seeds accumulated about 0.1%. Greater retention in roots can be attributed to reduced Cr transport from root to aerial parts of plant. Cr immobilization either by compartmentalization in vacuoles or retention in cation exchange sites of xylem parenchyma cells causes Cr accumulation in root, which is indeed a defensive strategy adapted by plant against metal toxicity [36]. Certain small sized proteins behave as natural chelates, bind as cation with the Cr ions, and inhibit its transport [30]. A reduction of Cr(VI) to low soluble form Cr(III) might be another possible reason of higher Cr accumulation in roots [22].

The bioaccumulation factor is the ratio of concentration of metal in shoot to that in soil. Bioaccumulation process is the ability of plant to convert and store the toxic metals into non-toxic or less toxic forms in various plant organs [37]. The values for BAF of hybrid Napier grass were greater than 1, which indicate the Cr(VI) tolerance capability of shoot of the plant. However, the TF values were lower than 1, which suggests the restricted ability of hybrid Napier grass to transport the Cr from root to shoot [27,38]. The hybrid Napier might be classified as Cr(VI) excluder because of its ability to effectively restrict the Cr transport and maintain relatively low Cr levels in shoot over a wide range of soil Cr(VI) contamination [39]. Moreover, the plant species would be applicable for Cr(VI) phytostabilization due to its BAF values > 1 and a relatively low TF value [7,40]. Furthermore, hybrid Napier grass is edible to animals; its ability to stabilize toxic metal in the root and limited transport to aerial (edible) parts of plant would prevent animal’s ingestion, restrict soil mobility, and consequently reduce transmission across the food chain.

EDTA application enhanced Cr uptake and accumulation in ET-plants when compared with NET-plants. In addition, Cr accumulation was comparatively higher in the shoot of ET-plant, which is due to the increased transfer factor in ET-plants as compared with NET-plants. Increased Cr uptake from soil and its transport from roots to aerial parts of the plants have been reported in Indian mustard (*Brassica juncea*) [4] and rapeseed (*Brassica. Napus* L.) [5]; and, in oats (*Avena sativa*), sesame (*Sesamum indicum*), Soyabean (*Glycine max*), okra (*Abelmoschus esculentus*), spinach (*Spinacia oleracea*), wheat, and sorghum [27]. EDTA either binds with Cr to form Cr-EDTA complex or increases the concentrations of soluble and exchangeable form of Cr by lowering soil pH and, thus, increases the bioavailability and facilitates the transport [24]. In the present study, the BAF and TF both increased with increasing Cr(VI) concentrations in soil and the EDTA application resulted in further enhancement of their values. Han et al. [4] and Ebrahimi et al. [7] reported an increase in both Cr accumulation factor and transfer factor with increasing Cr(VI) contamination in the soil and the EDTA addition led to a further increase in their values in common reed (*Phragmites australis* (Cav.) Trin. Ex Steudel) and Indian mustard.

### 3.3. Chlorophyll and Photosynthetic Efficiency

Consistent with our results, several studies have reported a decrease in chlorophyll with increasing Cr(VI) concentrations in soil. Two varieties of *Catharanthus roseus* (L.) i.e., C. *rosea* and C. *alba* grown in Cr(VI) contaminated soil for 30 days showed a reduction in chlorophyll by 10.56% and 4.72%, respectively [22]. The chlorosis effect of Cr(VI) might be due to its inhibitory effects on one or more enzymes that are involved in chlorophyll biosynthesis or it may be due to the damage of associated proteins [40,41].

Cr(VI) has been shown to impair photosynthesis either directly or indirectly by affecting one or more structural or functional components of photosynthetic machinery [23,30]. The efficiency of plant pigments to capture and convert light energy is represented by the Fv/Fm ratio, which is an excellent measurement of overall maximum quantum yield efficiency of photosystem-II (PSII) [28]. A reduction in the Fv/Fm ratio in our study suggests that Cr(VI) decreased the quantum efficiency of PSII photochemistry in hybrid Napier grass. Previous studies have reported a dose-dependent linear decrease in the Fv/Fm ratio with Cr(VI) concentrations between 0 to 300 μM in rice and wheat seedlings [23,28]. FV/Fm also specifically represents the overall efficiency of open PSII centers and photochemical quenching (qP) represents the number of open PSII centers [28,42]. In the present study, a concurrent decrease in qP and Fv/Fm suggests that Cr(VI) stress caused the shut down of some PSII centers as well as slowed down the efficiency of open PSII centers. Mathur et al. [28] reported that Cr(VI) stress reduced the active PSII centers count and thereby reduced its density in rice seedlings. In addition, lowered qP is associated with a simultaneous increase in qN [42]. In our study, maximum increase in qN by 49% reflects the dissipation of a huge amount of excitation energy under Cr(VI) stress [23]. Moreover, elevated qN inhibits NADPH and ATP utilization following the Cr(VI)-induced reduction of CO_2_ assimilation and ultimately leads to the impairment of photosynthetic electron transport (ETR) [42]. Consistently, decreased ETR by 17.6% in the present study reflects impaired electron flow. Obstructed electron flow from the reaction center to Quinone pool has been reported in rice seedlings under Cr(VI) stress [28]. One of the reasons of impaired electron flow in the present study may be the Cr(VI)-induced reduction in activity of water-splitting complex, as observed by a 29% decline in Fv/Fo. Similarly, Mathur et al. [28] observed a 30% decrease in Fv/Fo in wheat plantlets that were treated with 300 μM Cr(VI). Reduced Fv/Fo also represents structural damage, such as loss of thylakoid membranes, etc., in the chloroplast and it is a more reliable criterion in evaluating the photochemical activity [28,41].

With EDTA application, the damaging effects of Cr(VI) on photosynthetic activity were more pronounced, as shown by severe decrease in Fv/Fm, Fv/Fo, and ETR, by 31.63%, 134%, and 57.5%, respectively, and a increase in qN by 314% at Cr60 with EDTA addition (ET-plants) as compared with that of without EDTA addition (NET-plants). The toxic effects of Cr(VI) on photochemical parameters increase with the increase in amount of Cr in plant tissues [22]. The severe damage of photosynthetic activity in ET-plants might be attributed to greater Cr concentrations in leaves (shoot) of the plant, which was two times higher in the shoots of ET-plants as compared with NET-plants that might result from a EDTA-induced increase in the metal transport.

### 3.4. Elemental Status

Though, there was variable response of different Cr(VI) concentrations in soil on contents of N and S in hybrid Napier grass, but, in general, the Cr(VI) stress showed an increasing trend in N contents and decreasing trend in S content in root and shoot of the plant. Consistently, Wyszkowski and Radziemska [43] observed that soil contamination with Cr(VI) raised the N content by 21% and 37.5% in oats roots and straw, respectively, and the N accumulation was higher in the upper regions of maize and spring barley (*Hordeum vulgare* L.) [44]. The Cr(VI) stress produced variable effects on S contents in plants. S contents decreased in leaves, but increased in the stem and root of citrullus (*Citrullus vulgaris* cv. Ludhiana) cultivated in Cr(VI) contaminated soil [45]. Whereas, S content decreased in roots of *Brassica juncea* (L.) seedlings that were grown under Cr(VI) stress [46].

EDTA application further escalated the effects of Cr(VI) on contents of N and S in the plant. Generally, the contents of N increased and that of S decreased in root and shoot of the plant. Consistent with our findings, Zheng et al. [47] reported an increase in N content of *Lespedeza chinesis* and *L. davidii* with an increase in soil Pb concentrations and the addition of EDTA caused a further increase in the N-content of the plant. The mechanism that is involved in the reduction of S content in plant might be explained by the reason that Cr(VI) either competitively inhibits binding site and/or decrease sulfate transporter (BjST1) mRNA expression [46]. The Cr(VI)-induced S deficiency impairs S incorporation in some essential amino acids, thereby decreasing S-containing protein contents and eventually leading to stunted plant growth [48].

### 3.5. Oxidative Stress

In our study, the reduced tolerance was associated with simultaneous increase in oxidative stress in the plant. It has been suggested that Cr(VI)-induced impairment in biochemical pathways, such as photosynthesis and chlorophyll biosynthesis, are at least partly caused by oxidative stress. The Cr(VI) contamination causes an imbalance between reactive oxygen species (ROS), such as H_2_O_2_, and alters the activities of antioxidant enzymes and, thus, causes oxidative stress in leaves, shoot, and roots of the plants [49]. MDA is a product of lipid peroxidation that is used as an important marker of oxidative stress [36]. The increased MDA contents by 19.21% and 140.5% in root and shoot in our study signifies that Cr(VI) induced oxidative stress in the studied plant. Consistently, Upadhyay and Panda [50] observed an increase in MDA content by 182% and 140% in the root and shoot of water lettuce (*Pistia stratiotes* L.) at 10 mM Cr(VI) as compared with control. Similarly, Cr stress elevated the MDA contents in roots of cotton cultivars [51] and leaves of maize [49]. Concomitant with MDA content, we found an increase in activities of SOD by 43.29% and 123.37%, POD by 59.36% and 69.1%, and CAT by 27.17% and 135.82% in root and shoot of Cr(VI) stressed plants, respectively, when compared with control. Moreover, a dose-dependent increase in the activities of these antioxidants has been reported in roots of cotton cultivars [41] and leaves of maize [49].

The magnitude of oxidative stress induction was greater in the root and shoots of the plant with increasing Cr(VI) concentrations and further increased with the application of EDTA as compared with that of without EDTA application. The increase in enzyme activities suggested the induction of stress by Cr(VI), as there were no significant changes in enzyme activities among the controls (with and without EDTA). Similarly, Khan et al. [52] reported that the exposure of *Petunia hybrida* L. to Cr resulted in significantly higher antioxidant enzyme activity, which was enhanced with the increasing concentrations of Cr, and co-addition of EDTA along with Cr. The increasing toxic effects of Cr(VI) in combination with EDTA treatment might be related to increased toxicity with higher uptake and accumulation of Cr by EDTA treatment. Han et al. [4] reported that EDTA treatment increased the accumulation of Cr in *B. juncea*, which consequently resulted in growth retardation, reduction of the number of palisade, and spongy parenchyma cells in leaves, clotted depositions in the xylem, and phloem tissues of stems and roots.

## 4. Materials and Methods

### 4.1. Seed Collection and Cr(VI) Stock Solution Preparation

Seeds of hybrid Napier grass were purchased from Lizhiyuan seed company, Mianyang, China. Before sowing, the seeds were treated with 0.1% mercuric chloride (HgCl_2_) solution for 10 min. and then washed with distilled water to avoid any infection [27]. Stock solution of Cr(VI) (1000 mg L^−1^) was prepared by dissolving potassium dichromate (K_2_Cr_2_O_7_, 2.83 g) in deionized water (1000 mL). The stock solution was then appropriately diluted to get test solution of desired Cr(VI) concentrations.

### 4.2. Soil Collection and Seedling Growth

Soil was sampled from farmland far from Cr contamination source in Mianyang, China. The coordinates of the sampling points were determined while using a Macellel Model GPS and the locations of the sampling points are (coordinates, 31.550074 E–104.64034 5N). Five soil samples were collected from surface layer up to 30 cm depth and within a distance of 5 m surrounding the site to form a composite sample. After air drying, the samples were crushed to pass through a size of 2 mm sieve. Prior the experiment, a soil sample was analyzed for some physico-chemical properties, as presented in Table 4. The sieved soil was placed on waterproof tarpaulin and mixing Cr(VI) solution to obtain appropriate Cr(VI) (20, 40, and 60 mg kg^−1^) concentrations in soil. The soil was then allowed a minimum period of 30 days for stabilization. Soil without any amendment was used as control. Meanwhile, the seeds were sown about 1 cm deep in uncontaminated soil filled in small polyethylene bags for seedlings (one seed per one bag).

### 4.3. Experimental Design

Pot experiment was carried out in a completely randomized design (CRD) in naturally lit greenhouse at Southwest University of Science and Technology (SWUST), Mainyang, China, under ambient conditions. Pots (1 L in volume with dimensions; 11 cm height × 13 cm top diameter × 10 cm bottom diameter) were individually filled with 1 kg of uncontaminated and Cr(VI)-contaminated soil with varying Cr(VI) concentrations, respectively. The pots were equilibrated for 24 h and the seedlings of hybrid Napier grass (20 days older and uniform) were then transferred into pots. Experimental setup comprised of two; viz. non-EDTA-treated (NET) and EDTA-treated (ET) groups with four treatments (10 plants/treatment) in each group (Table 5). The treatments in NET group included Cr0 or control (plants grown in uncontaminated soil) and Cr20, Cr40, and Cr60 (plants grown in soil contaminated with Cr(VI) levels of 20, 40, and 60 mg kg^−1^, respectively) without any EDTA amendment. Whereas, in the ET group, the four treatments Cr0, Cr20, Cr40, and Cr60 followed similar pattern as in NET, amended with EDTA at the dose rate of 4 mM, applied once to the soil at 15 days after transfer of the seedlings. The plants were watered to 100% of field capacity (soil water content maintained at 41.9%). Each pot was placed in plastic saucers to collect leachates that were added back into pot soil regularly to minimize the loss of Cr(VI) and EDTA in the system. After seedlings transfer, the pot experiment lasted for 45 days; thereafter, the plants were harvested, sampled, and analyzed accordingly.

### 4.4. Growth Measurements

Five plants from each treatment were thoroughly rinsed and then cut into roots and shoots. After measurement of shoot height (cm), root length (cm), and leaf area (cm^2^) by using scale, the samples were then oven dried at 80 °C for 48 h and dry weights (g plant^−1^) were measured while using analytical weight balance.

### 4.5. Cr Analysis

The Cr concentrations in plant (shoot and root) and soil samples were determined through the acid digestion method, as described by Diwan et al. [36]. Briefly, the shoot (200 mg) and root (100 mg) samples were digested with a 5 mL mixture of HNO_3_ and H_2_O_2_ (4:1 *v/v*), whereas the soil (1000 mg) samples were digested with HNO_3_/HCl solution (3:1 *v/v*). The sample digestion was taken in a Teflon digestion vessel while using microwave-assisted digestion system (MDS-6G) for 15 min. to 120 °C, 15 min. to 190 °C, and 30 min. at 190 °C. The digested samples were finally diluted with deionized water to make a final volume up to 50 mL for subsequent Cr analysis through Inductively coupled plasma-optical emission spectrometry (ICP-OES; Varian 715-ES ICP-OES; Varian, Palo Alto, CA, USA). The Cr concentration and accumulation in root and shoot of the plant were calculated, as described by Farid et al. [53].

Cr accumulation (mg plant^−1^) = Cr concentration in organ (mg kg^−1^) × Dry weight of organ (kg)

### 4.6. Phytoremediation Potential

The bioaccumulation factor (BAF) and transfer factor (TF) of the metal were measured to determine the phytoremediation potential of the plant. BAF is the ratio of metal concentration in shoot to that in soil and TF is the ratio of metal concentration in plant shoot to that in roots [27]. BAF and TF were calculated as:

BAF = C_shoot_ (mg kg^−1^ DW)/C_soil_ (mg kg^−1^ DW)

TF = C_shoot_ (mg g^−1^ DW)/C_root_ (mg g^−1^ DW)

where, C_shoot_, C_root_, and C_soil_ are Cr concentrations in shoot, root, and soil, respectively.

### 4.7. Tolerance Index (TI)

The tolerance index (TI) was determined as the ratio between biomass (DW) of a Cr(VI) treated plant to that of a control plant [27], as follows:

TI = Biomass of the treated plants (g plant^−1^)/Biomass of the control plants (g plant^−1^)

### 4.8. Determination of Chlorophyll Content, Chlorophyll-α Fluorescence and Elemental Contents

The chlorophyll (Chl) content was non-invasively determined in the flag leaf while using a portable chlorophyll meter SPAD 502 (Minolta, Japan) [54]. The value of chlorophyll (Chl) content (mg m^−2^) was estimated from corresponding SPAD values by using the following equation:

Chl content (mg m^−2^) = 15.68 (SPAD units) − 209.03

Chlorophyll-α fluorescence was measured in five intact and healthy flag leaves through fluorometer (FMS2 from Hansatech Instruments Ltd., Norfolk, UK). After an adjustment period of 30 min. in the dark, the leaves were exposed to beam light as per the method adapted by Paiva et al. [41]. The maximum photosynthetic efficiency of PSII (ΦPSII = Fv/Fm), efficiency of water-splitting complex (Fv/Fo), and variable fluorescence (Fv = Fm − Fo) were calculated, where Fo and Fm denote the minimum and maximum fluorescence, respectively.

The contents (%) of nitrogen (N) and sulfur (S) in the root and shoot were analyzed through elemental analyzer (Vario EL cube; Elementar Analysensysteme, Langenselbold, Germany). The dried samples were ground into fine powder. The powdered samples (10 mg) were subjected to elemental analysis and the results were expressed in percentage (%).

### 4.9. Measurement of Oxidative Stress Parameters

The homogenized tissue (shoots and roots) samples were analyzed for oxidative stress parameters, as described by Anjum et al. [39]. Malondialdehyde (MDA, kit A003-1) level and activities of superoxide dismutase (SOD, EC 1.15.1.1, kit A001-1), peroxidase (POD, kit A084-3), and catalase (CAT, EC 1.11.1.6, kit A007-1) in plant tissues were quantified by using assay kits (Nanjing Jiancheng Bioengineering Institute, Nanjing, China). Briefly, the MDA content (µmol g^−1^ FW) was measured at 535 nm as an amount of MDA and thiobarbituric acid (TBA) mixture produced as a result of a reaction of MDA in samples with TBA. SOD activity was measured based on the inhibition of photochemical reduction of nitroblue tetrazolium (NBT) by O_2_•^−^ radicals. The activities of CAT or POD were calculated as the rate of H_2_O_2_ decomposition.

### 4.10. Statistical Analysis

The data were checked for normality and the homogeneity of variances and log-transformed to correct the deviations from these assumptions when needed. All of the measurements were tested by a two-way ANOVA by using the SPSS 16.0 for Windows statistical software package (SPSS Inc., Chicago, IL, USA). Post-hoc comparisons were tested while using Tukey’s test at a significance level of *p* < 0.05.

## 5. Conclusions

The results of the present study demonstrated that the contamination of soil with varying Cr(VI) concentrations increased Cr uptake and accumulation and negatively affected the growth of hybrid Napier grass in a dose-dependent manner. The Cr(VI) stress altered the levels of N and S, reduced chlorophyll content, and impaired photosynthetic machinery associated with reduced tolerance and increased oxidative stress. In addition, EDTA application enhanced Cr uptake and accumulation, along with more Cr accumulation in the shoot of ET-plants than that of NET-plants due to enhanced transport. Moreover, the phytotoxic effects of Cr(VI) increased in the presence of EDTA than without EDTA treatment. Though the Cr(VI) and EDTA stress reduced tolerance, but even at the highest Cr(VI) concentration, the plant could exhibited strong resistance, as evidenced by an increase in SOD, POD, and CAT activities. The hybrid Napier might be classified as Cr excluder because of its ability to maintain relatively low Cr levels in shoot by effective restriction of the Cr transport (TF < 1). Moreover, due to its BAF values > 1 and TF < 1, this plant species would be applicable for Cr phytostabilization. Furthermore, hybrid Napier grass is edible to animals; its ability to limit the toxic metal to aerial (edible) parts of plant would prevent animal’s ingestion, restrict soil mobility, and consequent transmission across the food chain. However, this species needs to be further explored to understand the molecular mechanism of tolerance and remediation potential of Cr, as well as other heavy metals with various chelant amendments, so that the plant can be best utilized in the field of phytoremediation.

## Figures and Tables

**Table 1:** Natural and average Cr concentration in soils of different regions of the world.

Cr Concentration (mg kg^−1^)	Parameter	Region	Reference
50–600	Back ground concentration	-	Ma and Hooda [8]
5–3000	Back ground concentration	India	Shanker et al. [9]
2–60	Natural concentration	Turkey	Isıklı et al. [10]
10–50	Natural concentration	-	Adriano [11]
100	Average concentration	West Indies	Mandal and Voutchkov [12]
59.5	Average concentration	Poland	Kabata-Pendias [13]
22	Average concentration	Sweden	Eriksson [14]
58	Average concentration	Japan	Takeda et al. [15]
54	Average concentration	USA	Burt et al. [16]
94.8	Average concentration	Finland	Salminen et al. [17]

Adapted from Shahid et al. [2].

**Table 2:** Influence of various Cr(VI) concentrations in soil on Cr concentration and Cr accumulation in root and shoot, and Transfer factor (TF) and Bioaccumulation factor (BAF) of hybrid Napier grass with and without EDTA application.

Soil Cr(VI) Conc. (mg kg^−1^)		Cr Concentration (mg kg^−1^)		Cr Accumulation (µg Plant^−1^)		TF	BAF
		Root	Shoot	Root	Shoot		
0	Without EDTA	2.45 ± 0.89 ^g^	1.48 ± 0.45 ^f^	0.69 ± 0.34 ^g^	1.77 ± 0.6 ^f^	-	-
	With EDTA	1.22 ± 0.02 ^g^	2.38 ± 1.52 ^f^	0.34 ± 0.08 ^g^	2.69 ± 1.4 ^f^	-	-
20	Without EDTA	374.6 ± 2.4 ^d^	22.2 ± 1.41 ^g^	94.37 ± 7.75 ^c^	20.29 ± 1.6 ^g^	0.06 ± 0.005 ^e^	1.11 ± 0.07 ^f^
	With EDTA	233.7 ± 0.6 ^f^	46.97 ± 3.15 ^e^	50.01 ± 1.55 ^e^	32.66 ± 2.8 ^e^	0.2 ± 0.014 ^e^	2.35 ± 0.16 ^c^
40	Without EDTA	473.6 ± 1.7 ^b^	64.6 ± 0.84 ^d^	105 ± 2.49 ^a^	46.66 ± 1.9 ^c^	0.14 ± 0.002 ^g^	1.61 ± 0.02 ^d^
	With EDTA	261 ± 0.92 ^e^	114.5 ± 5.6 ^b^	49.05 ± 1.51 ^f^	62.71 ± 4.3 ^b^	0.44 ± 0.022 ^c^	2.86 ± 0.14 ^a^
60	Without EDTA	504.7 ± 0.7 ^a^	73.35 ± 4.06 ^c^	101 ± 3.44 ^b^	41.4 ± 2.65 ^d^	0.15 ± 0.008 ^f^	1.22 ± 0.07 ^e^
	With EDTA	467 ± 1.96 ^c^	157.21 ± 12 ^a^	79.45 ± 3.6 ^d^	65.9 ± 7.79 ^a^	0.34 ± 0.03 ^d^	2.62 ± 0.2 ^b^
**Statistical Effect**							
Cr(VI) conc.		***	***	***	***	***	***
EDTA		***	***	***	***	***	***
Cr(VI) conc. × EDTA		***	***	***	ns	***	***

Values (means ± SE, *n* = 5) with dissimilar letters show significant difference at *p* < 0.05 from Tukey’s test, between different Cr(VI) doses within each treatment, and between two treatments (with and without EDTA), respectively, in the columns. *** *p* ≤ 0.001. ns: non-significant, Cr conc.: soil Cr(VI) concentration effect; EDTA: EDTA effect and Cr conc. EDTA: Cr(VI) conc. × EDTA interaction effect.

**Table 3:** Effects of various Cr(VI) concentrations in soil on tolerance indices (TI) of root and shoot of hybrid Napier grass with and without EDTA application.

Soil Cr(VI) Conc. (mg kg^−1^)	Shoot TI		Root TI	
	Without EDTA	With EDTA	Without EDTA	With EDTA
0	1	1	1	1
20	0.76 ± 0.024 ^a^	0.61 ± 0.021 ^b^	0.89 ± 0.047 ^a^	0.77 ± 0.02 ^b^
40	0.6 ± 0.022 ^c^	0.48 ± 0.02 ^d^	0.79 ± 0.054 ^b,c^	0.68 ± 0.03 ^c^
60	0.47 ± 0.017 ^e^	0.37 ± 0.022 ^f^	0.71 ± 0.02 ^c,d^	0.61 ± 0.026 ^d^
**Statistical Effect**				
Cr conc.	***		***	
EDTA	***		***	
Cr × EDTA	ns		**	

Values (means ± SE, *n* = 5) with dissimilar letters show significant difference at *p* < 0.05 from Tukey’s test, between different Cr(VI) doses within each treatment, and between two treatments (with and without EDTA), respectively, in the columns. ** *p* < 0.001; *** *p* ≤ 0.001. ns: non-significant, Cr conc.: soil Cr(VI) concentration effect; EDTA: EDTA effect and Cr conc. EDTA: Cr(VI) conc. × EDTA interaction effect.

**Table 4:** Physico-chemical properties of the background soil.

Properties	Determined Value
Sand (%)	1.4 ± 0.052
Silt (%)	23.9 ± 0.56
Clay (%)	74.7 ± 0.84
pH	6.0 ± 0.06
Texture class	Silty clay
Electrical conductivity (mS m^−1)^	0.71 ± 0.28
Total Carbon (C, %)	0.132 ± 0.002
Hydrogen (H, %)	0.381 ± 0.007
Nitrogen (N, %)	0.057 ± 0.002
Sulfur (S, %)	0.003 ± 0
Total chromium (Cr_total_, mg kg^−1^)	0.0104 ± 0

**Table 5:** Experimental design showing groups and treatments.

Treatments	Groups							
	NET-Plants				ET-Plants			
	Cr0	Cr20	Cr40	Cr60	Cr0	Cr20	Cr40	Cr60
Soil Cr(VI) conc. (mg k^−1^)	0	20	40	60	0	20	40	60
EDTA (mM)	-	-	-	-	4	4	4	4

EDTA: Ethylene diamine tetra acetic acid; NET-plants: Non-EDTA-treated plants; ET-plants: EDTA-treated plants.
